# Puerarin alleviates apoptosis and inflammation in kidney stone cells via the PI3K/AKT pathway: Network pharmacology and experimental verification

**DOI:** 10.1111/jcmm.70180

**Published:** 2024-10-27

**Authors:** Yuexian Xu, Hu Liang, Xike Mao, Zhenyu Song, Xudong Shen, Defeng Ge, Yang Chen, Bingbing Hou, Zongyao Hao

**Affiliations:** ^1^ Department of Urology The First Affiliated Hospital of Anhui Medical University Hefei China; ^2^ Institute of Urology Anhui Medical University Hefei China; ^3^ Anhui Province Key Laboratory of Urological and Andrological Diseases Research and Medical Transformation Anhui Medical University Hefei China

**Keywords:** apoptosis, kidney stone, network pharmacology, puerarin

## Abstract

Puerarin(PUE), an isoflavonoid extracted from Pueraria root, has anti‐apoptotic effects. The objective of this research is to examine the impact of PUE on renal apoptosis and inflammation resulting from renal calculi and to elucidate its mechanism. The approach of network pharmacology and molecular docking was employed to discover potential targets and pathways of PUE. An animal model of calcium oxalate crystal deposition by intraperitoneal injection of glyoxylate and a model of COM‐induced human renal tubular epithelial cells (HK2) were used to investigate the pharmacological mechanisms of PUE against apoptosis and inflammation. We used haematoxylin–eosin (H&E) and Periodic Acid‐Schiff staining (PAS) to assess the effect of PUE on crystal deposition and damage. The mechanism of PUE was elucidated and validated using Western blotting, histology and immunohistochemical staining. Network pharmacology findings indicated that the PI3K/AKT pathway plays a crucial role in PUE. We experimentally demonstrate that PUE alleviated COM‐induced changes in apoptotic proteins, increased inflammatory indicators and changes in oxidative stress‐related indicators in HK2 cells by activating the PI3K/AKT pathway, reduced serum creatinine and urea nitrogen levels in mice caused by CaOx, alleviated crystal deposition and damage, and alleviated apoptosis, oxidative stress and inflammation. Puerarin attenuates renal apoptosis and inflammation caused by kidney stones through the PI3K/AKT pathway.

## 1 INTRODUCTION

The prevalence of kidney stones, a frequent urological condition, has been on the rise for the last 20 years.^1^, ^2^, ^3^ Despite notable advancements in less invasive surgical methods, the rate of recurrence within a span of 5 years following the initial operation remains at 50%,^4^ resulting in substantial socioeconomic and medical burdens.^5^ The main component of kidney stones is calcium oxalate (CaOx) crystals, which account for up to 80% of all kidney stones.^6^ CaOx causes kidney damage mainly because of apoptosis, autophagy, inflammation, fibrosis and so on.^7^, ^8^, ^9^, ^10^ Considering this, the discovery of novel therapeutic targets holds immense theoretical importance and practical value in the treatment and prevention of kidney stones.

The method of treating kidney stones with herbal medicine has a long history and profound cultural heritage. With the deepening of scientific research, more and more studies have confirmed that herbal medicine has significant efficacy in the treatment of kidney stones. Its unique advantage lies in its smaller side effects and high safety, providing a more gentle and effective treatment approach for kidney stone patients.^11^, ^12^, ^13^ PUE, derived from Pueraria Mirifica, is a bioactive compound abundant in flavonoids, peptides and other bioactive substances.^14^ An increasing amount of pharmacological research has shown that PUE exhibits a range of biopharmacological properties. PUE has been shown to have anti‐inflammatory, anti‐injury and anti‐apoptotic effects in vitro in a large number of experiments.^15^, ^16^, ^17^, ^18^, ^19^ Not only in vitro experiments, but also in vivo experiments have fully verified its efficacy. PUE attenuates neurological deficits apoptotic pathways in subarachnoid haemorrhage mice.^20^ PUE suppresses inflammation and alleviates pain symptom in osteoarthritic mice.^21^ PUE protects against myocardial ischemia in rats.^22^ The effect of PUE on the kidney has also been demonstrated by a large number of in vivo experiments. The renoprotective effect of PUE has been demonstrated in diabetic nephropathy, renal fibrosis and cisplatin‐induced acute kidney injury.^23^, ^24^, ^25^, ^26^ Nonetheless, limited research has been conducted on the safeguarding impact of PUE against CaOx‐induced renal damage.

Network pharmacology, as a new research methodology, has been successful in revealing multiple targets and complex mechanisms of drugs in various diseases. Using a network pharmacology method, we examined the possible targets and pathways through which PUE can reduce renal injury caused by CaOx. To validate our hypothesis, we conducted experiments both in vivo and in vitro.

## 2 MATERIALS AND METHODS

### 2.1 Network pharmacology

GeneCards (Giftes≥5) was used to search through the Swiss target prediction database (www.swisstargetprediction.ch) for potential targets associated with kidney stones. Venny tool draw the Venn diagram, kidney stones and PUE‐related targets. Protein–protein interaction(PPI) between potential targets were evaluated using the STRING database (https://cn.string‐db.org/). Using Cytoscape 3.7, we built targets and kidney stones of PUE potential targets for shared between PPI, through the CytoHubba plug‐in filtered on key targets. The DAVID database (david.abcc.ncifcrf.gov) was used for enrichment analysis of the intersecting genes. Molecular docking was performed using AutoDock.

### 2.2 Chemicals and reagents

PUE and LY294002 (PI3K/AKT inhibitor) were purchased from TargetMol (Shanghai, USA). Glyoxylate and calcium oxalate monohydrate (COM) were obtained from Sigma (Germany). TNF‐α (26405‐1‐AP), IL‐1β (16765‐1‐AP), IL‐6 (21865‐1‐AP), AKT (10176‐2‐AP), p‐AKT (Ser 473) (28731‐1‐AP), PI3K (20584‐1‐AP), Bax (50599‐2‐Ig), Bcl‐2 (26593‐1‐AP) and Caspase3 (19677‐1‐AP) were obtained from Peprotech (Wuhan, China). p‐PI3K (#AF3242) was obtained from Affinity Biosciences(Jiangsu, China). Using the Cell Counting Kit‐8 (CCK‐8) sourced from Beyotime (Nanjing, China) and ELISA kits from Solarbio (China), we assayed various oxidative stress markers—specifically, MDA, SOD, LDH and GSH in the cell homogenates derived from different treatment cohorts.

### 2.3 Cell culture and treatment

The human kidney cell line HK‐2 was obtained from the Institute of Basic Medical Sciences of the Chinese Academy of Medical Sciences. These cells were cultured in an environment containing 10% fetal bovine serum and a DMEM/F12 mixed medium, maintained at 5% carbon dioxide and 95% humidity for routine cultivation. Before stimulating with COM (100 μg/mL), we exposed the HK‐2 cells to different concentrations of PUE (1, 2, 4, 8, 16, 32, 64, 128 μM) or LY294002 (10 μM) for 30 min. Afterwards, they were transferred to a new environment containing 0.5% fetal bovine serum and a DMEM/F12 mixed medium for overnight cultivation.

According to the manufacturer's instructions, Lipofectamine 3000 (Invitrogen, MA, USA) was used to transfect HK2 cells with siRNA‐PI3K (GenePharma, Shanghai, China). Subsequent experiments were performed after the cells were incubated for 24 h. The target sequences are as follows: PI3K, 5'‐GAAAGGAGGAAATAACAAA‐3′.

### 2.4 Cell viability determination

CCK‐8 was utilized to assess cell viability. In summary, HK2 cells were seeded in 96‐well dishes and prior to stimulation with COM (100 μg/mL), they were treated with varying amounts of PUE for 24 h. Next, 10 microliters of CCK‐8 solution was introduced into every well and incubated for a period of 60 to 90 min. The manufacturer's instructions (Multiskan MK3, Thermo, United States) were followed to analyse the optical density at 492 nm.

### 2.5 Animals and experimental design

We conducted rigorous and cautious animal experiments. In this experiment, we carefully selected male C57BL/6J mice aged between 6 and 8 weeks as the subjects to ensure the accuracy and reliability of the experimental results. It's worth mentioning that we strictly adhere to animal ethics norms and this experiment has been officially authorized by the Animal Ethics Committee of Anhui Medical University (numbered LLSC20232250). This committee is committed to ensuring that all scientific research activities involving animals follow the highest ethical standards, fully respecting and protecting the rights and interests of animals. Their authorization provides important guarantees for the compliance and morality of our experiment. To create a mouse model for CaOx renal calcium deposition, a daily intraperitoneal injection of 100 mg/kg glyoxylate was administered and the mice were euthanized after 7 days of injection. Mice received PUE(10, 50 and 100 mg/kg) via oral administration, 12 h prior to glyoxylate injection, followed by daily administration. We anaesthetised the mice with Sodium barbiturate (50 mg/kg) (Sigma, 11,715) intraperitoneally, collected blood samples from their orbits for analysis and killed them after they had been fasted and water deprived for 12 h. The blood that was acquired underwent testing for Cr and BUN in accordance with the instructions provided by the manufacturer, while the renal tissue was regularly embedded in paraffin and examined for molecular objectives. ImageJ software was used to determine the percentage of CaOx crystal deposition in each kidney section.

### 2.6 Tubular injury determination

In order to assess the damage to tubules, we conducted PAS staining using a PAS staining kit according to the established protocol. Subsequently, we invited three experienced pathologists to collaboratively determine the tubule damage score based on the degree of loss of brush border, tubule dilation and atrophy, as well as the formation of intraluminal casts. Regarding tubular lesions, we have established the following grading criteria^27^: 0 (indicating normal), 1 (≤10%), 2 (11%–25%), 3 (26%–50%), 4 (51%–75%) and 5 (≥76%).

### 2.7 Histology and immunohistochemical staining

Paraffin‐embedded sections of mouse kidneys were prepared using standard histopathology techniques, including 4% paraformaldehyde fixation, dehydration, waxing, embedding and sectioning (4 μm). Kidney sections were then stained with HE and PAS reagent kits using standard protocols (Solarbio, Beijing, China). First, Immunohistochemistry was performed by renal biopsy antigen repair and then endogenous peroxidase activity was blocked. The antibodies were incubated for 30 min at a temperature of 4°C. Tissue samples were stained using the DAB (3,3′‐diaminobenzidine) staining method. After staining was completed, all observed images were captured and recorded using an Olympus IX83 microscope (made in Japan).

### 2.8 Western blot analysis

Using 10% SDS‐PAGE technology, separate 30 micrograms of tissue lysate and cell samples. The separated samples of cells mixed with tissue lysate are then transferred to a nitrocellulose (NC) membrane using electrophoretic transfer. Block the NC membrane for 2 h using tris‐buffered saline containing 0.05% Tween 20 and 5% non‐fat milk. Cells are incubated with a specific primary antibody for 8 h. Subsequently, the cells are co‐incubated with a secondary antibody at 37°C for 90 min. Images are captured and analysed using the Licor/Odyssey infrared imaging system. Finally, the grey values of each band are quantified using ImageJ software.

### 2.9 TUNEL staining

Following the guidelines provided by Solarbio (Beijing, China), we conducted the TUNEL assay on 5 μm tissue sections and HK2 cells. After completing the assay, we first rinsed the slides twice with phosphate‐buffered saline and then stained them with DAPI (Bioworld). Finally, we carefully examined the slides under a fluorescence microscope (Olympus IX83, Japan).

### 2.10 Enzyme‐linked immunosorbent assay (ELISA)

We used an ELISA kit (provided by Solarbio, Beijing, China) to detect the levels of superoxide dismutase (SOD), malondialdehyde (MDA), glutathione (GSH) and lactate dehydrogenase (LDH). The entire experimental process strictly followed the operating guidelines of the cellular ELISA kit.

### 2.11 Measurement of Superoxide Anion and ROS Level

The concentrations of superoxide anion and ROS were assessed employing the dihydroethidium (DHE) assay kit (Beyotime Biotechnology, S0063, Shanghai, China) and the 2′,7′‐dichlorofluorescein (DCF) kit (Beyotime Biotechnology, S0033S, Shanghai, China). The cellular samples were then inspected and captured using a fluorescence microscope (Olympus IX83, Japan).

### 2.12 Statistical analysis

Statistical analyses were conducted using GraphPad Prism 8.3.0 (GraphPad Software, San Diego, CA, USA). The presented data represent the mean with standard deviation. Variations among distinct groups were analysed via one‐way ANOVA.

## 3 RESULTS

### 3.1 Network pharmacology analysis

**FIGURE 1:**
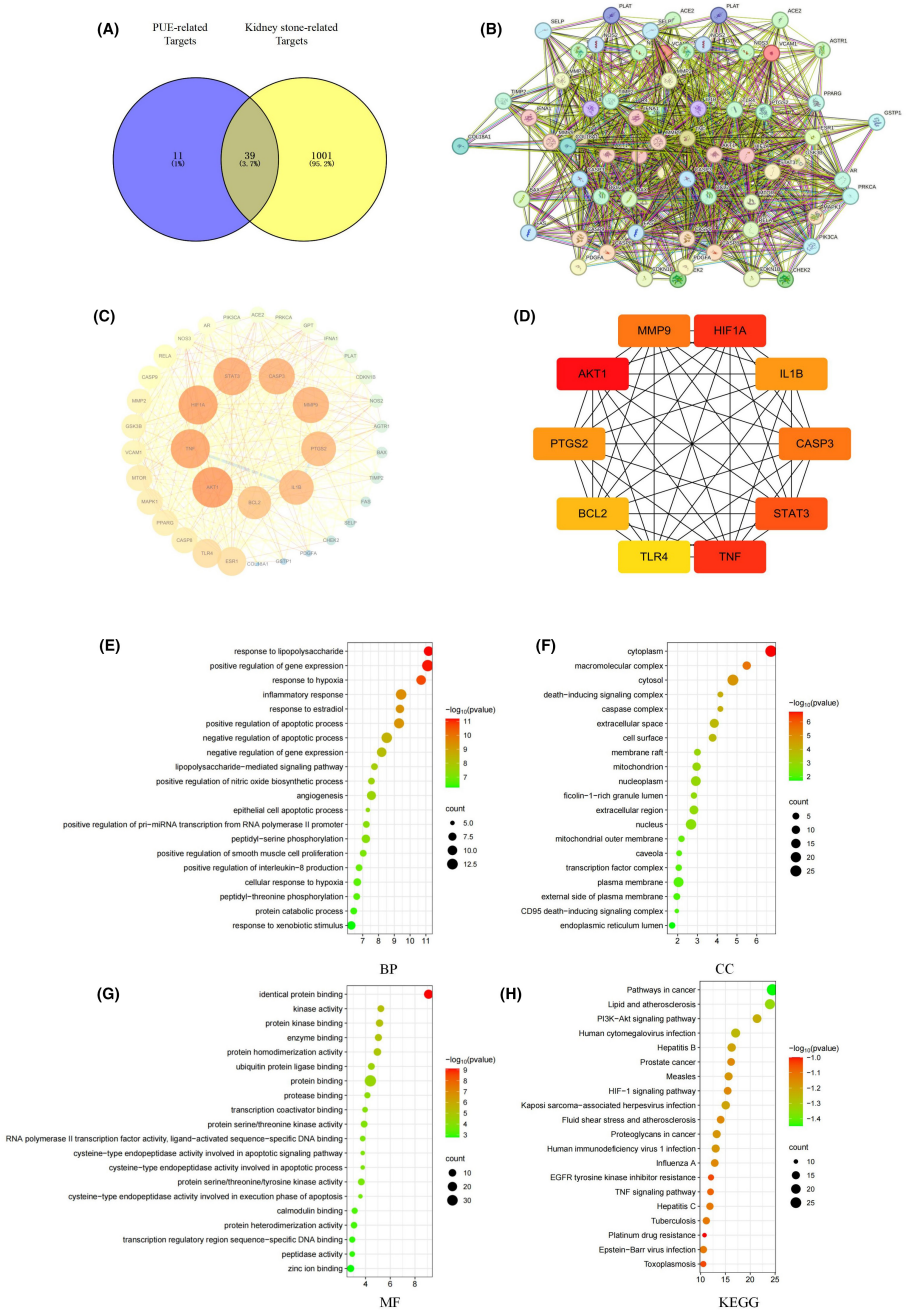
Venn diagram and PPI network and Enrichment analysis. (A) Venn diagram of the intersection target between PUE and Kidney stone. (B) PPI network of targets generated using STRING 11.0.(C) PPI network of targets genetrated useing Cytoscape 3.7. (D) Top 10 targets of PPI network. (E) Top 20 cellular components in Gene Ontology. (F) Top 20 biological processes in Gene Ontology. (G) Top 20 molecular functions in Gene Ontology. (H) Top 20 pathways in the Kyoto Encyclopedia of Genes and Genomes (KEGG). The abscissa indicates gene proportion, the ordinate shows pathway name, bubble size represents the number of targets in the pathway and colour indicates *p*‐value.

After cross‐referencing kidney stone targets with PUE targets, we successfully identified 39 common drug‐disease targets (Figure 1A). Leveraging the STRING online platform, we constructed an interaction network for these shared targets (Figure 1B). Subsequently, we imported the network diagram and data information obtained from the platform into Cytoscape 3.7.0 software for in‐depth analysis and based on this, we built a PPI network (Figure 1C). To determine the key nodes in the network, we used the cytoHubba plug‐in to screen for hub genes (Figure 1D). Detailed information on these core regulatory genes is listed in Table 1. We performed enrichment analysis using 39 shared drug‐disease targets of the GO and KEGG pathways. The top of each part is shown in the bubble graph. Based on the analysis of biological processes (BP), the identified targets are linked to the inflammatory response, promotion of apoptotic process, inhibition of apoptotic process and apoptosis of epithelial cells (Figure 1E). Cellular component(CC) analysis showed that the target distribution in the nucleus, plasma membrane, cytoplasm, cytosol and nucleoplasm(Figure 1F). Molecular function (MF) show the connections and functions between these targets, such as protein binding, cysteine−type endopeptidase activity involved in apoptotic signalling pathway, cysteine−type endopeptidase activity involved in apoptotic process(Figure 1G). The KEGG enrichment analysis results indicated a strong association between the shared targets and the PI3K/AKT signalling pathway (Figure 1H).

**TABLE 1:** Basic topological properties of core regulatory genes.

Geen symbol	Average shortest PathLength	Betweenness centrality	Closeness centrality	Neighbourhood connectivity
IL‐1β	1.13157895	0.02327975	0.88372093	24.24242424
STAT3	1.07894737	0.03130638	0.92682927	23.6
CASP3	1.10526316	0.02567058	0.9047619	24.20588235
PTGS2	1.13157895	0.02462452	0.88372093	24.12121212
MMP9	1.10526316	0.03072048	0.9047619	23.79411765
TLR4	1.23684211	0.01227401	0.80851064	25.86206897
HIF1A	1.05263158	0.04019006	0.95	23.19444444
AKT1	1.02631579	0.04827868	0.97435897	22.7027027
TNF‐α	1.05263158	0.04119749	0.95	23.02777778
BCL2	1.15789474	0.02057588	0.86363636	24.5625

### 3.2 Molecular docking study

**FIGURE 2:**
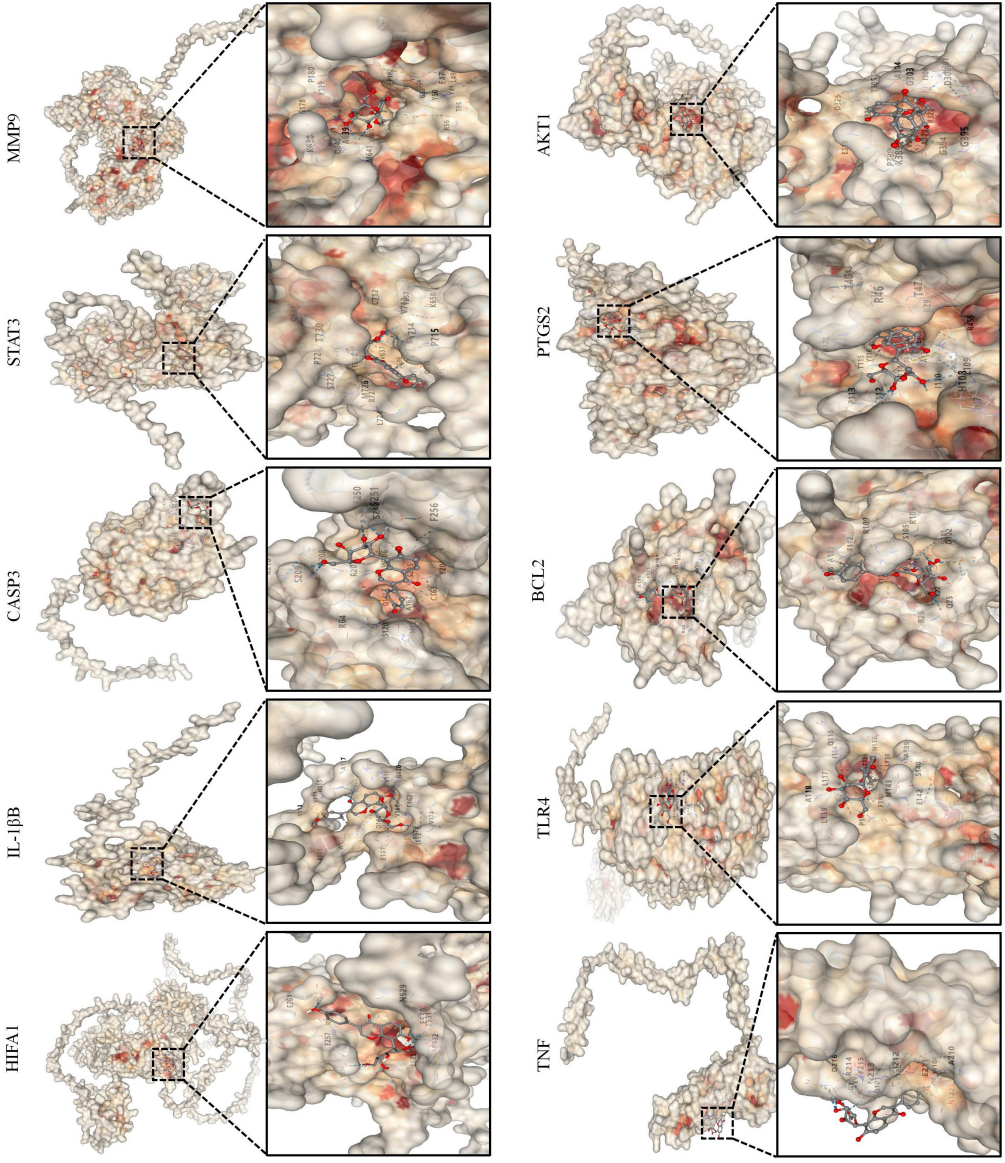
Molecular docking. Molecular models of the binding of PUE with MMP9, STAT3, CASP3, IL‐1β, HIF1A, AKT1, PTGS2, BCL2, TLR4, TNF‐α.

Based on the selected HUB gene, we used molecular docking technology to predict the binding affinity between PUE and its target protein and visualize the results (Figure 2). Relevant research indicates that when the docking score is <0 kcal/mol, the ligand can automatically bind to the receptor, and when the docking score is < −5.0 kcal/mol, a better docking between the two can be achieved.^28^ The binding energies of action were shown in Table 2. The docking results showed that the best binding energy of a compound and target was −8.5 kcal/mol, and the worst was −6.4 kcal/mol. This indicates that PUE can effectively bind to the 10 most crucial drugdisease targets very well.

**TABLE 2:** Docking score of PUE with each core target.

Gene name	Dockingscore (kcal/mol)
HIF1	−7.3
IL‐1β	−7.8
CAS3	−7.4
SAT3	−8.3
TNF‐α	−6.4
TLR4	−7.2
BCL2	−8.1
PTGS2	−8.5
MMP9	−8.0
AKT1	−7.8

### 3.3 PUE attenuated COM crystal‐induced HK2 cell injury, inflammation and oxidative stress

**FIGURE 3:**
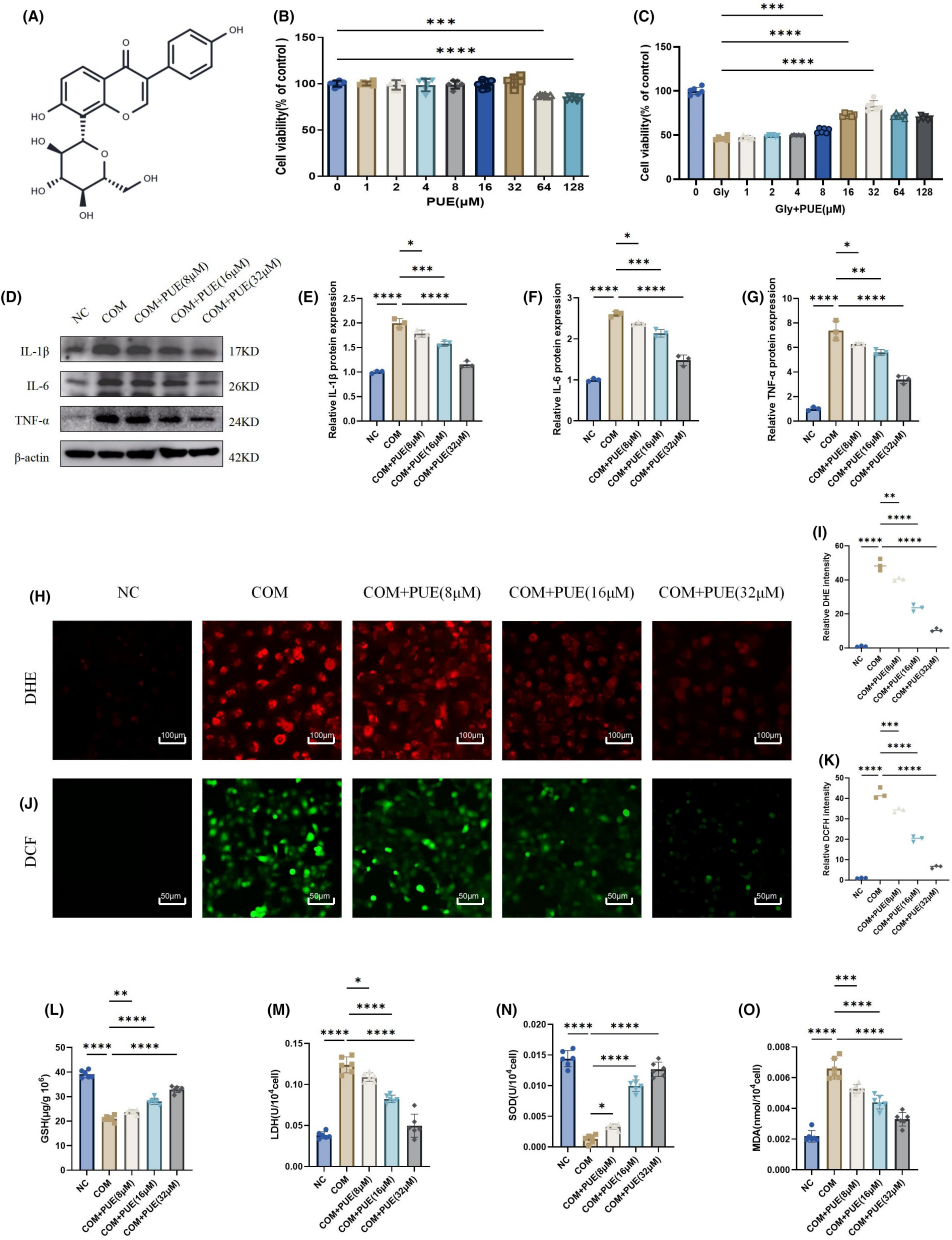
PUE prevents COM crystal‐induced cell injury and inflammation. (A) The molecular formula of PUE. (B) We tested the cytotoxicity of PUE on HK2 cells at different concentrations using the CCK‐8 assay. (C) The PUE pretreatment of HK2 cells exposed to COM crystals restored their viability. (D–G) Western blot and quantitative analysis of IL‐1β, IL‐6 and TNF‐α. (H–K) The levels of superoxide anion and reactive oxygen species in HK‐2 cells were detected using DHE and DCF staining. (L) GSH content (μg/10^6^ cell). (M) LDH content (U/10^4^cell). (N) SOD activity (U/10^4^cell). (O) MDA content (nmol/10^4^cell). Data are shown as the mean ± S.E.M. ****p < 0.0001, ***p < 0.001, **p < 0.01, *p < 0.05.

The molecular formula of PUE is shown in Figure 3A. Initially, the impact of PUE on HK2 cells was assessed for its cytotoxicity, and the outcomes from CCK‐8 tests indicated that HK2 cell viability remained largely unaffected by PUE concentrations lower than 64 μM. Furthermore, we conducted additional research on the impact of PUE on the damage caused to HK2 cells by COM crystals(Figure 3B). The CCK‐8 experiments indicated that exposure to COM crystals resulted in a notable reduction in the viability of HK2 cells, which was alleviated by PUE(Figure 3C), PUE concentrations of 8, 16 and 32 μM were more effective in improving HK2 cell viability, so we used these three concentrations in subsequent cell experiments. We then found that COM crystals induced a significant increase in IL‐1β, IL‐6 and TNF‐α, while PUE significantly reduced the inflammatory response(Figure 3D–G). To determine whether PUE causes oxidative stress in HK‐2 cells by inhibiting COM, we further examined oxidative stress‐related indicators. By measuring COM crystal‐induced ROS generation by fluorescence and confocal microscopy, we found that PUE treatment partially attenuated ROS(Figure 3H–K). By assaying MDA, SOD, LDH and GSH we further confirmed that PUE significantly reduced the level of COM‐induced oxidative stress as the dose increased(Figure 3L–O).

### 3.4 In vitro, PUE counteracts the impact of COM on the PI3K/AKT signalling pathway and apoptosis

**FIGURE 4:**
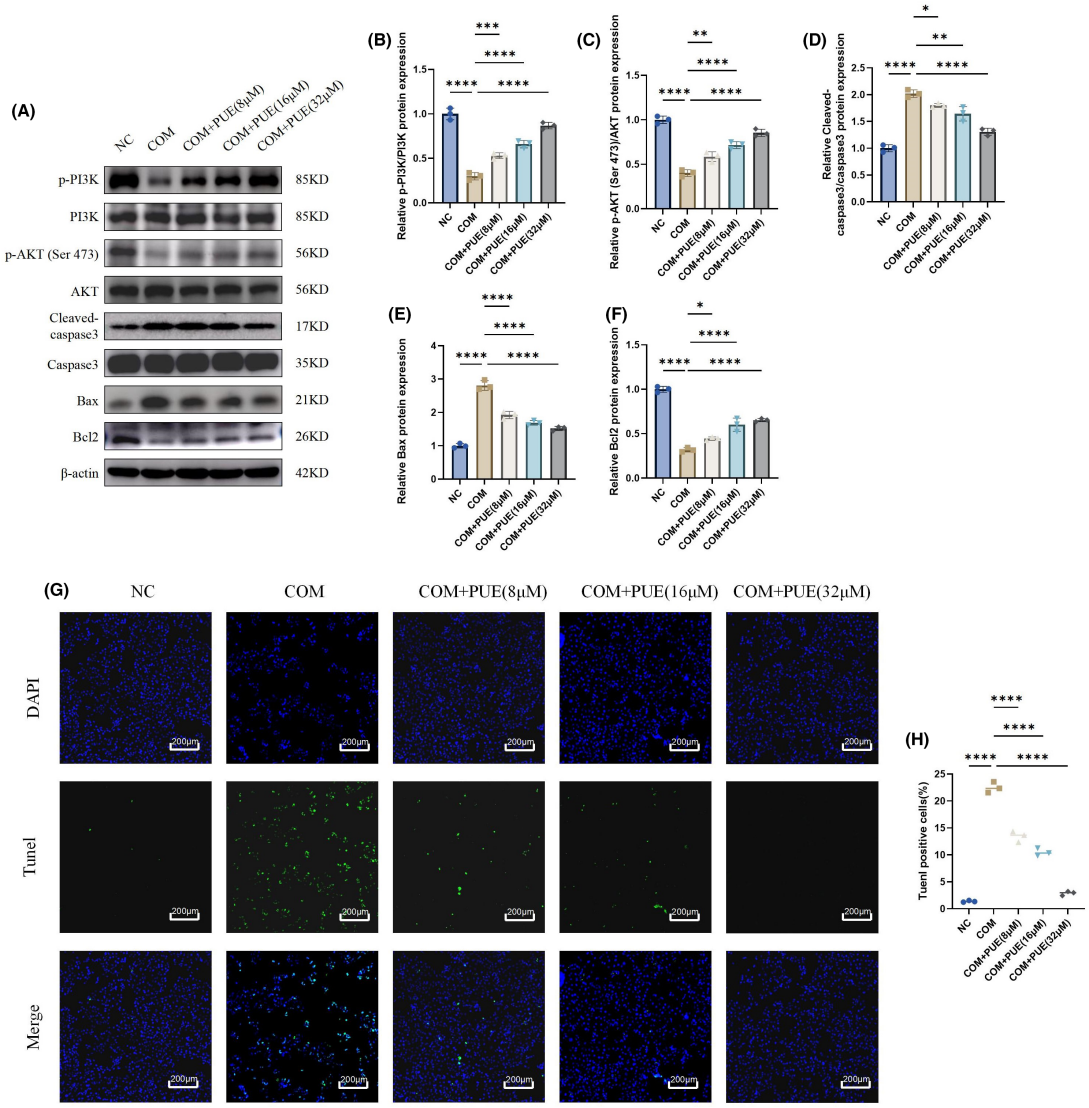
PUE attenuates COM crystal‐induced cell apoptosis. (A–F) Western blot and quantitative analysis of p‐PI3K, PI3K, p‐AKT (Ser 473), AKT, Cleaved‐caspase3, caspase3, Bax and Bcl2. (G, H) HK‐2 cells were stained with TUNEL for detection of apoptosis. The staining shows green fluorescence when the cells are in the process of dying. Data are shown as the mean ± S.E.M. ****p < 0.0001, ***p < 0.001, **p < 0.01, *p < 0.05.

PUE has been repeatedly shown to have anti‐apoptotic effects.^29^, ^30^, ^31^ The GO analysis described previously indicates that PUE likely exerts its protective effects by alleviating apoptosis. In the KEGG analysis, we found that drug‐disease targets were significantly enriched in the PI3K/AKT pathway. The PI3K/AKT signalling pathway has been confirmed to play a significant role in the formation of kidney stones.^32^, ^33^ In this study, we investigated whether PUE can reduce apoptosis in HK2 cells through the PI3K/AKT signalling pathway in vitro using COM. Initially, it was observed that p‐PI3K and p‐AKT (Ser 473) exhibited a notable decrease in the COM group when compared to the control group. However, this decrease was counteracted by PUE. Additionally, the COM group displayed significant alterations in Cleaved‐caspase3, Bax and Bcl2 levels, which were also reversed by PUE in a dose‐dependent manner (Figure 4A–F). This suggests that PUE can reverse the apoptosis produced by HK2 cells caused by COM. We then used TUNEL staining to show that treatment with PUE ameliorated COM‐induced apoptosis in HK‐2 cells(Figure 4G,H). We fully demonstrated that PUE alleviated COM‐induced apoptosis in HK2 cells.

### 3.5 PUE treatment for renal calcification is a CaOx disease mouse model has a protective effect on inflammation and damage

**FIGURE 5:**
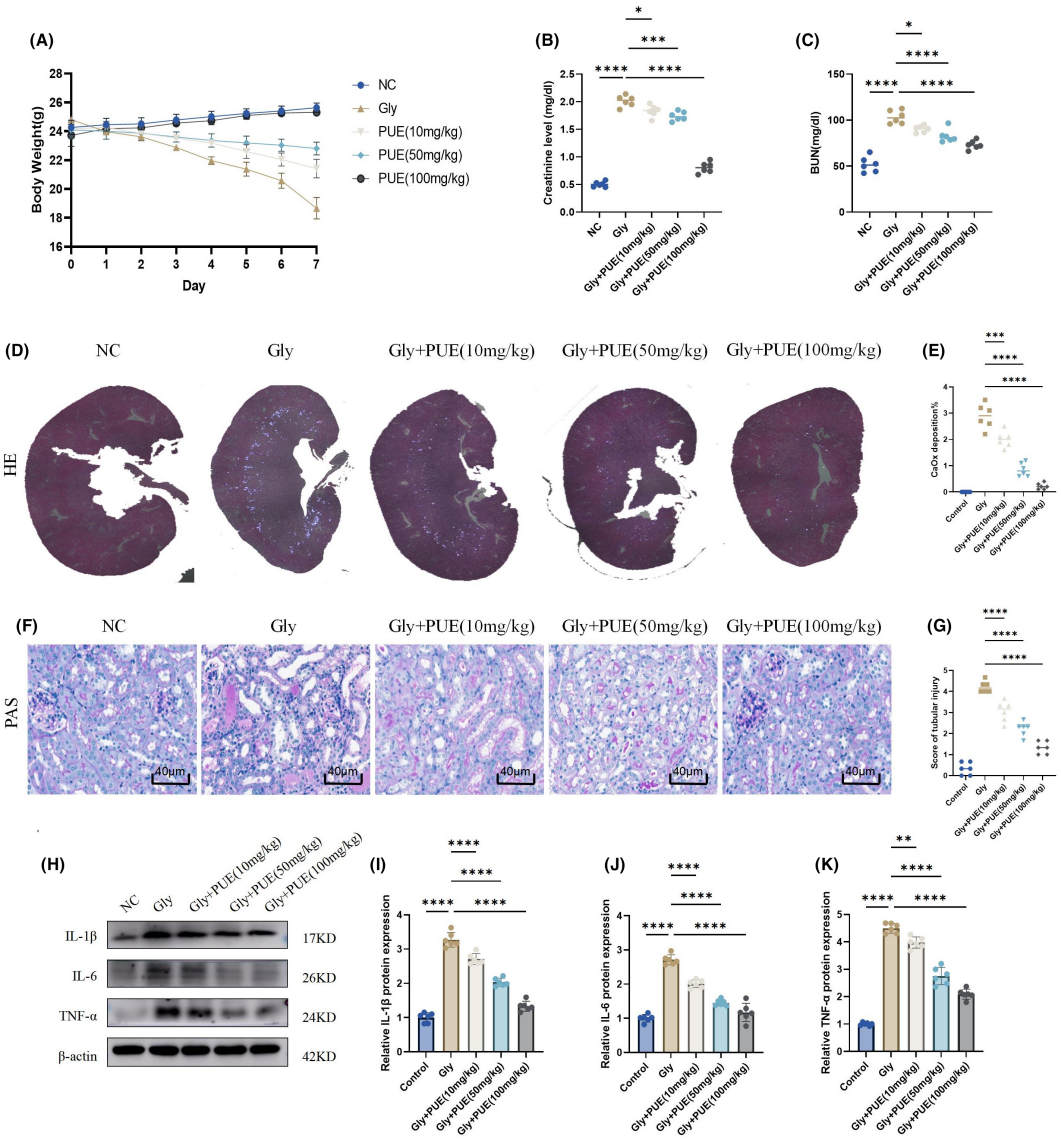
PUE protected against CaOx crystal‐induced renal injury and inflammation in mice. (A) Change in weight. (B, C) Serum Cr and BUN in the mice with CaOx crystalline nephropathy with or without PUE administration. (D, E) Polarized light microscopy was used to visualize CaOx crystal deposition in kidney sections and quantify it as a percentage. (F, G) PAS staining was used to illustrate and score tubular injury. (H‐K) Western blot and quantitative analysis of IL‐1β, IL‐6 and TNF‐α. Data are shown as the mean ± S.E.M. ****p < 0.0001, ***p < 0.001, **p < 0.01, *p < 0.05.

Subsequently, we examined the defensive impact of PUE on in vivo renal calcinosis caused by CaOx. Mice were given PUE at doses of 10, 50 and 100 mg/kg/d after being pretreated with gavage 12 h prior to the injection of glyoxylate. The findings indicated that PUE mitigated the decrease in body weight in a mouse model of CaOx renal calcinosis (Figure 5A) and significantly decreased elevated levels of serum Cr and BUN in a mouse model of CaOx renal calcinosis (Figure 5B,C). Additionally, there was a decline in the formation of CaOx crystals in the renal system, as depicted in Figure 5D,E. PAS staining showed that PUE treatment also partially reversed renal tubular injury (Figure 5F,G). Western blot analysis demonstrated that PUE effectively decreased the protein levels of IL‐1β, IL‐6 and TNF‐α induced by CaOx, as depicted in Figure 5H–K.

### 3.6 In vivo, PUE counteracts the impact of CaOx on the PI3K/AKT signalling pathway and apoptosis

**FIGURE 6:**
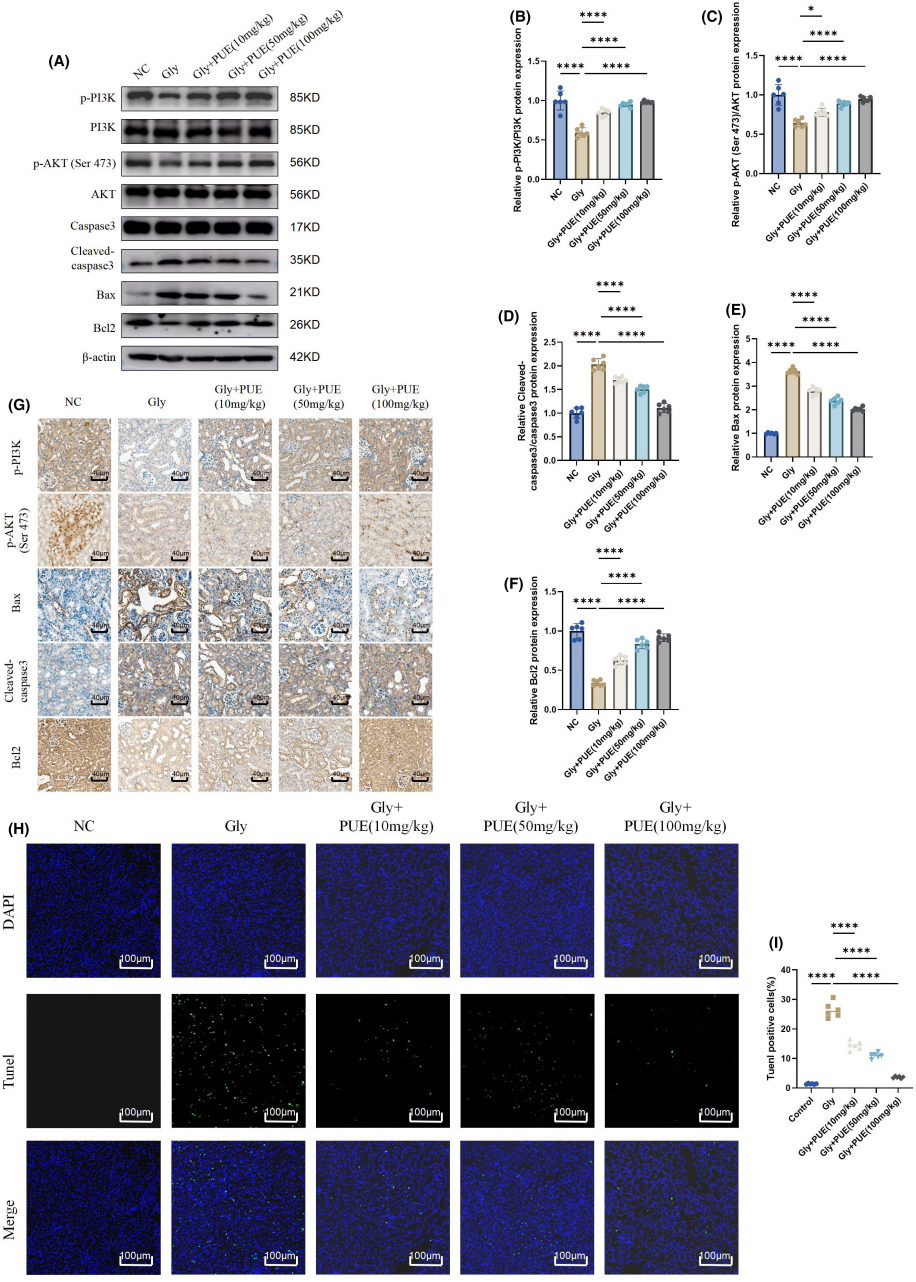
PUE protected against CaOx crystal‐induced renal apoptosis in mice. (A–F) Western blot and quantitative analysis of p‐PI3K, PI3K, p‐AKT (Ser 473), AKT, Cleaved‐caspase3, caspase3, Bax and Bcl2. (G) Representative immunohistochemistry images and quantitative analysis of p‐PI3K, PI3K, p‐AKT (Ser 473), AKT, Cleaved‐caspase3, caspase3, Bax and Bcl2. (H, I) Renal were stained with TUNEL for detection of apoptosis. Data are shown as the mean ± S.E.M. ****p < 0.0001, *p < 0.05.

We then observed in vivo the changes in p‐PI3K, p‐AKT (Ser 473) and apoptosis‐related proteins induced by PUE on CaOx. By Western blotting and IHC we found that PUE attenuated the inhibitory effect of CaOx on the renal PI3K/AKT signalling pathway and alleviated the CaOx‐induced changes in cleaved‐caspase3, Bax and Bcl2 levels (Figure 6A–F). Immunohistochemistry yielded consistent results(Figure 6G). The results of TUNEL staining also showed that PUE ameliorated CaOx‐induced renal apoptosis(Figure 6H,I).

### 3.7 PI3K inhibitor reverses the effects of PUE to alleviate COM damage, inflammation and oxidative stress in HK2 cells

**FIGURE 7:**
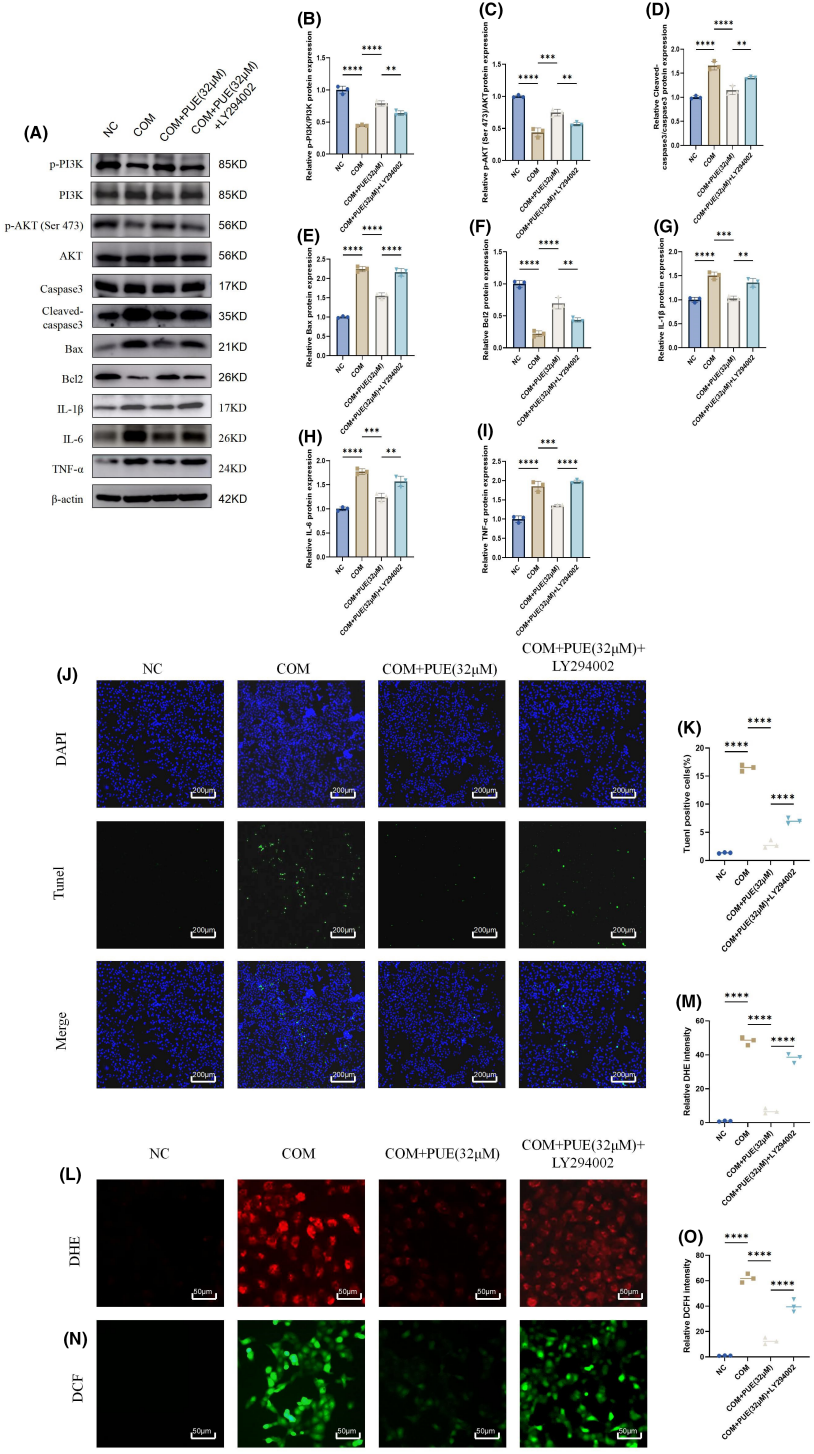
PUE exerted protective effects by PI3K/AKT pathway. (A–I) Western blot analysis of p‐PI3K, PI3K, p‐AKT (Ser 473), AKT, Cleaved‐caspase3, caspase3, Bax, Bcl2, IL‐1β, IL‐6 and TNF‐α in each group. (J, K) HK‐2 cells were stained with TUNEL for detection of apoptosis. (L‐O) The levels of superoxide anion and reactive oxygen species in HK‐2 cells were detected using DHE and DCF staining. Data are shown as the mean ± S.E.M. ****p < 0.0001, ***p < 0.001, **p < 0.01.

To provide additional confirmation of PUE's ability to mitigate the impact of COM on inflammation, apoptosis and oxidative stress in HK2 cells through modulation of the PI3K/Akt signalling pathway, we employed PI3K inhibitor(LY294002). For the concentration of PUE we used 32uM, which was the most effective in the in vitro experiments described above. Western blot analysis revealed that LY294002 counteracted the anti‐inflammatory and anti‐apoptotic impacts of PUE (Figure 7A–I). Subsequently by TUNEL staining we found that LY294002 reversed the anti‐apoptotic effect of PUE(Figure 7J,K). Then for further proof, we further examined oxidative stress‐related indicators and found that LY294002 reversed PUE to reduce COM‐induced superoxide anion and ROS levels in HK2 cells(Figure 7L,O).

### 3.8 The influence of PI3K siRNA on the alleviation of COM‐induced apoptosis, inflammation and oxidative stress in HK2 cells by PUE

**FIGURE 8:**
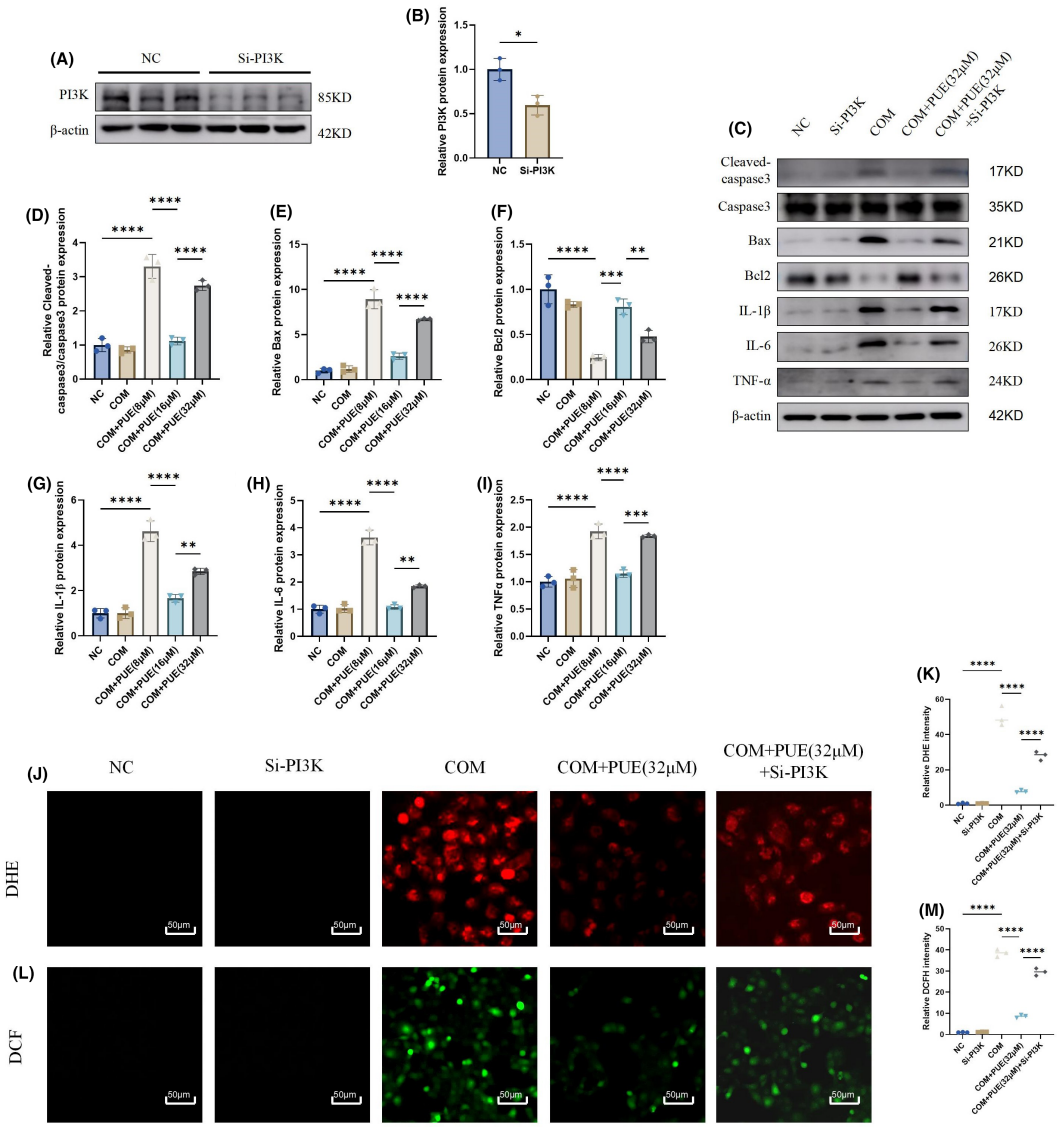
The influence of PI3K siRNA on the alleviation of COM‐induced apoptosis, inflammation and oxidative stress in HK2 cells by PUE. (A, B) Detection of transfection efficiency of Si‐PI3K by Western blot. (C–I) Western blot analysis of Cleaved‐caspase3, caspase3, Bax, Bcl2, IL‐1β, IL‐6 and TNF‐α in each group. (J–M) The levels of superoxide anion and reactive oxygen species in HK‐2 cells were detected by DHE and DCF. Data are shown as the mean ± S.E.M. ****p < 0.0001, ***p < 0.001, **p < 0.01, *p < 0.05.

In order to provide more reliable evidence for PUE's alleviation of COM‐induced apoptosis, inflammation and oxidative stress in HK2 cells via the PI3K/AKT pathway, we transfected Si‐PI3K (Figure 8A,B). We found through Western blotting that after reducing PI3K expression, the alleviating effects of PUE on COM‐induced apoptosis and inflammation in HK2 cells were largely reversed (Figure 8C–I). Furthermore, by detecting DHE and DCF, we discovered that the antioxidant stress effect of PUE was also diminished due to the transfection with Si‐PI3K (Figure 8J–M).

## 4 DISCUSSION

Network pharmacology has been extensively employed to comprehend the intricate mechanisms of medication treatment. Network pharmacology allows rapid screening of drug targets, prediction of pathways of action and systematic analysis of drug‐disease interactions.^34^ PUE is a major isoflavonoid extracted from the Chinese herb Pueraria Mirifica. Pueraria Mirifica, originating from Southeast Asia, has been utilized for countless centuries as sustenance, remedy and animal feed. Moreover, it is among the earliest plants employed in ancient China.^35^ Through the utilization of network pharmacology, we anticipated the capability of PUE in addressing kidney stones and subsequently validated its efficacy via both in vitro and in vivo experiments. This study revealed a strong correlation between apoptosis and the potential target of PUE in the context of kidney stones. The PI3K/AKT pathway may be associated with the anti‐apoptotic mechanism of PUE, as indicated by enrichment analysis of the constructed PPI network. Phosphoinositide 3‐kinase (PI3K), a lipid kinase, has a crucial function in both normal and abnormal cellular processes. PI3K controls the regulation of proliferation, differentiation, programmed cell death and migration by activating protein kinase B (PKB or AKT).^36^, ^37^ The role of PI3K/AKT pathway in inflammation, oxidative stress and apoptosis in kidney disease has been demonstrated by a large number of studies and plays an important role in kidney disease.^38^, ^39^ The PI3K/AKT pathway has been confirmed to be closely related to kidney stones.^40^, ^41^ PUE has also been shown to exert its pharmacological effects through the PI3K/AKT pathway.^42^, ^43^ Later we also predicted the affinity of PUE and core target using molecular docking. Therefore, we believe that PUE can alleviate CaOx‐induced renal apoptosis through the PI3K/AKT pathway and proved our hypothesis through in vitro and in vivo experiments.

Reactive oxygen species (ROS), collectively referred to as free radicals, consist of atoms or molecules possessing unpaired electrons. These highly reactive species are crucial in the modulation of signalling molecules.^44^ In addition, they have the ability to chemically alter and breakdown proteins, lipids, carbohydrates and nucleotides. Prior research has indicated the existence of ROS buildup in renal tissues containing calcium oxalate crystal deposits, indicating that ROS might play a role in the advancement and growth of calcium oxalate kidney stone disorder.^45^ In addition, clinical studies additionally validated that the serum of patients with stones exhibited a reduced level of antioxidant enzymes in comparison to the normal group. This indicates that individuals with stones have a diminished antioxidant capacity in their bodies and their levels of antioxidant enzymes are lower compared to the normal group.^46^ The levels of SOD and CAT activity, as well as the levels of MDA and GSH in tissues, are representative of the oxidative status of the tissues, lipid peroxidation and cellular injury.^47^, ^48^, ^49^ Therefore, we responded to the level of oxidative stress by measuring MDA, GSH, SOD and LDH in cells. Studies have reported differential proteins associated with inflammatory cells and processes in CaOx stone patients and controls.^50^ We found that PUE significantly reduced the level of CaOx‐induced oxidative stress and inflammatory responses in vivo and in vitro by a series of methods. PUE not only successfully alleviated the renal function of mice with a reduction in serum creatinine and urea but also attenuated the deposition and damage of calcium oxalate crystals.

Since the PI3K/AKT pathway is a key hub where PUE acts, we found by western blotting and HIC that PUE activated the PI3K/Akt pathway. The involvement of apoptosis in kidney disease is substantiated by human studies that reveal the activation of pro‐apoptotic pathways within kidney tissue. Additionally, preclinical data suggest that the protective effect is achieved through the disruption of genuine pro‐apoptotic proteins.^51^ The ability of CaOx to cause apoptosis in the kidney has been well documented.^52^, ^53^, ^54^ In our study, we confirmed our idea by testing the anti‐apoptotic effect of PUE by various means in vivo and in vitro.

To further confirm that PUE acts through the PI3K/AKT pathway, we applied an inhibitor of PI3K (LY294002).^55^ We found that the anti‐inflammatory, antioxidative stress and antiapoptotic effects of PUE were greatly reduced after inhibiting the PI3K/AKT pathway. Then, we reduced the expression of PI3K by transfecting Si‐PI3K and found that after decreasing PI3K expression, the anti‐apoptotic, anti‐inflammatory and antioxidant stress effects of PUE were largely reversed. This further proves our idea.

## 5 CONCLUSION

Using the method of network pharmacology, we initially examined the mechanism of action of PUE in treating kidney stones. The PI3K/AKT pathway was identified by network pharmacology as the mechanism through which PUE exerts its anti‐apoptotic effects. Following that, the in vivo and in vitro experiments confirmed the preventive and therapeutic capabilities of PUE in treating kidney stones. The scientific foundation for the impact of PUE on kidney stones and the practical use of PUE is established.

## AUTHOR CONTRIBUTIONS

**Yuexian Xu:** Conceptualization (equal); data curation (equal); resources (equal); software (equal); writing – original draft (equal). **Hu Liang:** Software (equal). **Xike Mao:** Software (equal). **Zhenyu Song:** Software (supporting). **Xudong Shen:** Resources (supporting). **Defeng Ge:** Data curation (supporting). **Yang Chen:** Investigation (supporting). **Bingbing Hou:** Writing – review and editing (supporting). **Zongyao Hao:** Funding acquisition (equal); writing – review and editing (equal).

## FUNDING INFORMATION

This study was supported by the National Natural Science Foundation of China (82070724), (82370768).

## CONFLICT OF INTEREST STATEMENT

The authors declare no conflicts of interest.

## CONSENT

All authors supported the publication of the manuscript.

## Data Availability

All data generated or analysed during this study are included in this article. Further inquiries can be directed to the corresponding author.
