# Correction to “Puerarin Alleviates Apoptosis and Inflammation in Kidney Stone Cells Via the PI3K/AKT Pathway: Network Pharmacology and Experimental Verification”

**DOI:** 10.1111/jcmm.70230

**Published:** 2024-12-27

Y. Xu, H. Liang, X. Mao, et al., “Puerarinalleviates Apoptosis and Inflammation in Kidney Stone Cells Via the PI3K/AKT Pathway: Network Pharmacology Andexperimental Verification,” *Journal of Cellular and Molecular Medicine* 28 (2024): e70180, https://doi.org/10.1111/jcmm.70180.

In the article, there were errors in Figure 3J and Figure 7. The COM + PUE (16 μM) set of images in Figure 3 J and NC set of images in Figure 7J were incorrect. The correct Figures 3J and Figure 7 are shown below. The authors confirmed that these errors do not affect the findings and conclusion of the article.
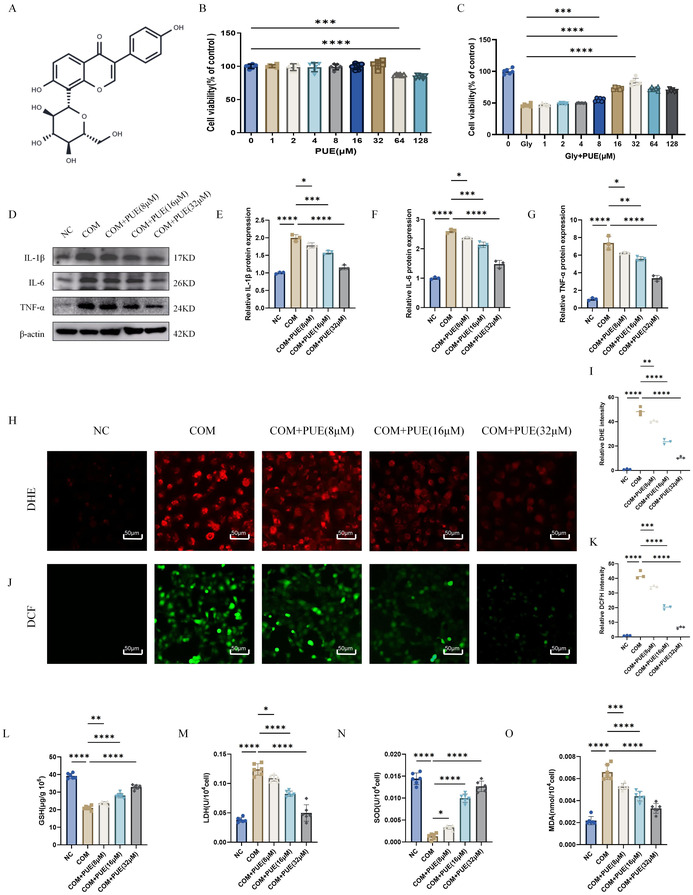
 
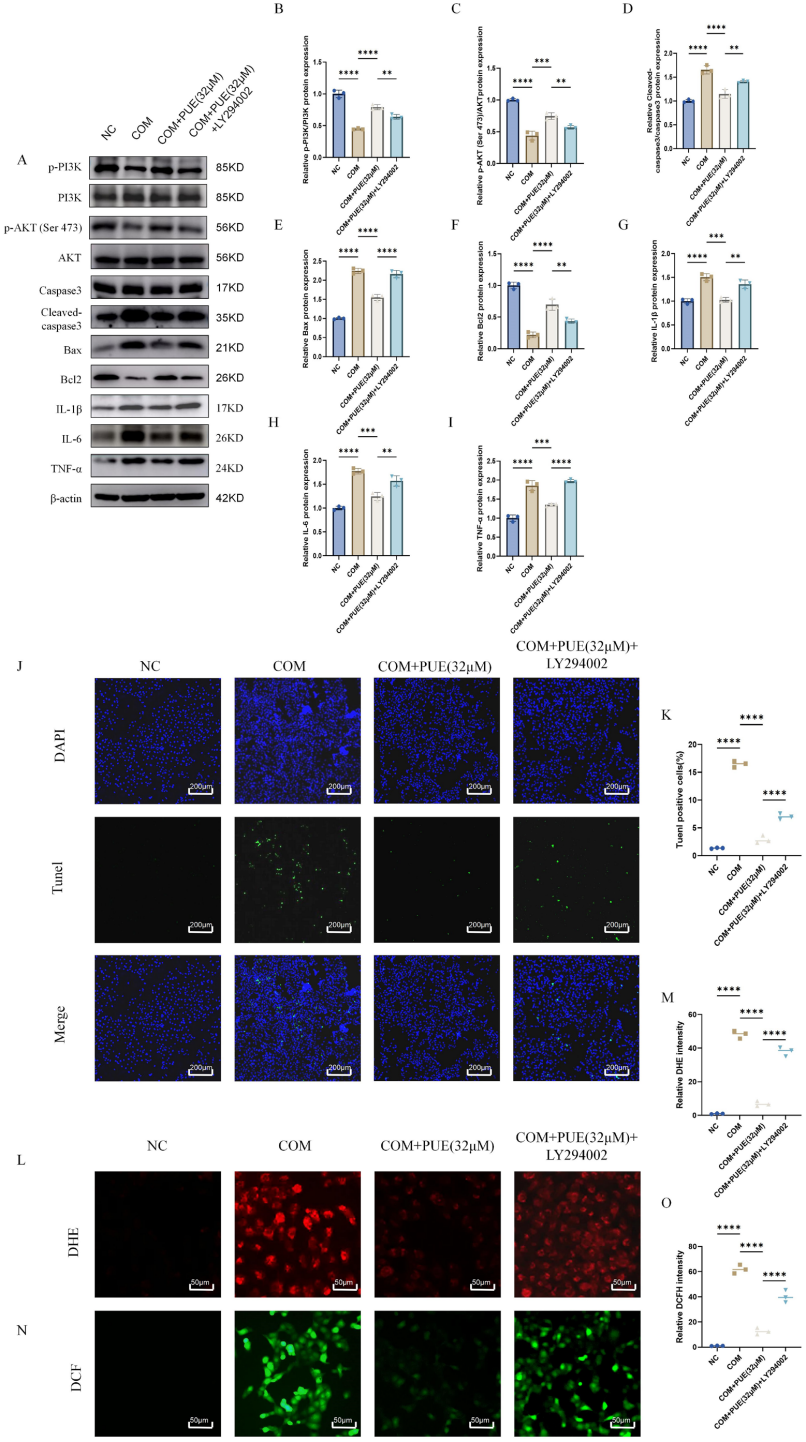


We apologise for these errors.

